# Advanced biological ageing predicts future risk for neurological diagnoses and clinical examination findings

**DOI:** 10.1093/brain/awad252

**Published:** 2023-07-25

**Authors:** Christopher E McMurran, Yunzhang Wang, Jonathan K L Mak, Ida K Karlsson, Bowen Tang, Alexander Ploner, Nancy L Pedersen, Sara Hägg

**Affiliations:** Department of Medical Epidemiology and Biostatistics, Karolinska Institutet, Stockholm SE 171 77, Sweden; Department of Clinical Neurosciences, University of Cambridge, Cambridge CB2 0QQ, UK; Department of Medical Epidemiology and Biostatistics, Karolinska Institutet, Stockholm SE 171 77, Sweden; Department of Medical Epidemiology and Biostatistics, Karolinska Institutet, Stockholm SE 171 77, Sweden; Department of Medical Epidemiology and Biostatistics, Karolinska Institutet, Stockholm SE 171 77, Sweden; Department of Medical Epidemiology and Biostatistics, Karolinska Institutet, Stockholm SE 171 77, Sweden; Department of Medical Epidemiology and Biostatistics, Karolinska Institutet, Stockholm SE 171 77, Sweden; Department of Medical Epidemiology and Biostatistics, Karolinska Institutet, Stockholm SE 171 77, Sweden; Department of Medical Epidemiology and Biostatistics, Karolinska Institutet, Stockholm SE 171 77, Sweden

**Keywords:** biological ageing, twin study, examination

## Abstract

Age is a dominant risk factor for some of the most common neurological diseases. Biological ageing encompasses interindividual variation in the rate of ageing and can be calculated from clinical biomarkers or DNA methylation data amongst other approaches. Here, we tested the hypothesis that a biological age greater than one's chronological age affects the risk of future neurological diagnosis and the development of abnormal signs on clinical examination.

We analysed data from the Swedish Adoption/Twin Study of Aging (SATSA): a cohort with 3175 assessments of 802 individuals followed-up over several decades. Six measures of biological ageing were generated: two physiological ages (created from bedside clinical measurements and standard blood tests) and four blood methylation age measures. Their effects on future stroke, dementia or Parkinson's disease diagnosis, or development of abnormal clinical signs, were determined using survival analysis, with and without stratification by twin pairs.

Older physiological ages were associated with ischaemic stroke risk; for example one standard deviation advancement in baseline PhenoAge_Phys_ or KDMAge_Phys_ residual increased future ischaemic stroke risk by 29.2% [hazard ratio (HR): 1.29, 95% confidence interval (CI) 1.06–1.58, *P* = 0.012] and 42.9% (HR 1.43, CI 1.18–1.73, *P* = 3.1 × 10^−4^), respectively. In contrast, older methylation ages were more predictive of future dementia risk, which was increased by 29.7% (HR 1.30, CI 1.07–1.57, *P* = 0.007) per standard deviation advancement in HorvathAge_Meth_. Older physiological ages were also positively associated with future development of abnormal patellar or pupillary reflexes, and the loss of normal gait.

Measures of biological ageing can predict clinically relevant pathology of the nervous system independent of chronological age. This may help to explain variability in disease risk between individuals of the same age and strengthens the case for trials of geroprotective interventions for people with neurological disorders.

## Teaser

People seem to age at different rates, and this can be measured using clinical data or marks on DNA. In a cohort of 802 Swedish twins followed up over several decades, McMurran *et al.* show that having a biological age greater than one's chronological age can affect future risk of suffering a stroke or developing dementia.

## Introduction

Ageing—the loss of biological function with the passage of time—is a dominant risk factor for some of the most common diseases in the neurology clinic.^1-3^ Whilst chronological age (time since birth) captures much of this risk, there can also be wide variation in age-associated outcomes between individuals of the same chronological age. The concept of biological ageing offers an explanation for some of this heterogeneity, theorizing that individuals age at different rates: age acceleration or deceleration. Approaches taken to quantify biological age have combined various biomarkers (including bedside clinical measurements, routine laboratory bloods tests and methylation data) with different algorithms (such as predicting chronological age directly versus predicting mortality risk, or tissue-generic versus tissue-specific approaches).^4-10^ Besides explaining some of the chronological age-adjusted variance in morbidity, biological age provides a conceptual framework to intervene on age-related diseases by using geroprotective treatments to slow biological ageing.^11,12^

Several previous studies have assessed biological age in the context of age-associated neurological conditions.^4^ For example, there is some evidence for accelerated blood methylation age amongst groups of patients with dementia,^13,14^ Parkinson's disease^15^ and multiple sclerosis.^16^ Outside of a specific disease context, a higher blood epigenetic age is also associated with age-related CNS structural changes (such as loss of white matter integrity)^17^ and functional decline (such as loss of general cognitive ability).^18,19^ Similarly, biological age estimated from routine blood tests can predict dementia more accurately than chronological age.^20^

Despite several established associations between biological age and neurological health and disease, there is currently a paucity of longitudinal studies. Multiple assessments of biological age over time (including prior to diagnosis) may help to distinguish cause and effect. For example: does accelerated biological age drive neurological disease (such as dementia, stroke or Parkinson's disease), or does the condition itself cause physiological changes resulting in age acceleration? Outside of a specific disease context, certain abnormalities on bedside neurological examination also become more common with advancing chronological age, e.g. problems with vibration sense, pupillary and tendon reflexes and gait.^21–24^ These findings likely represent underlying subclinical pathology as well as contributing to disability and increased risk of falls in older individuals.^25^ Their relationship to biological age has not been previously described and could offer insight into general mechanisms of neurological decline that occur in so-called ‘healthy’ ageing.

**Figure 1:**
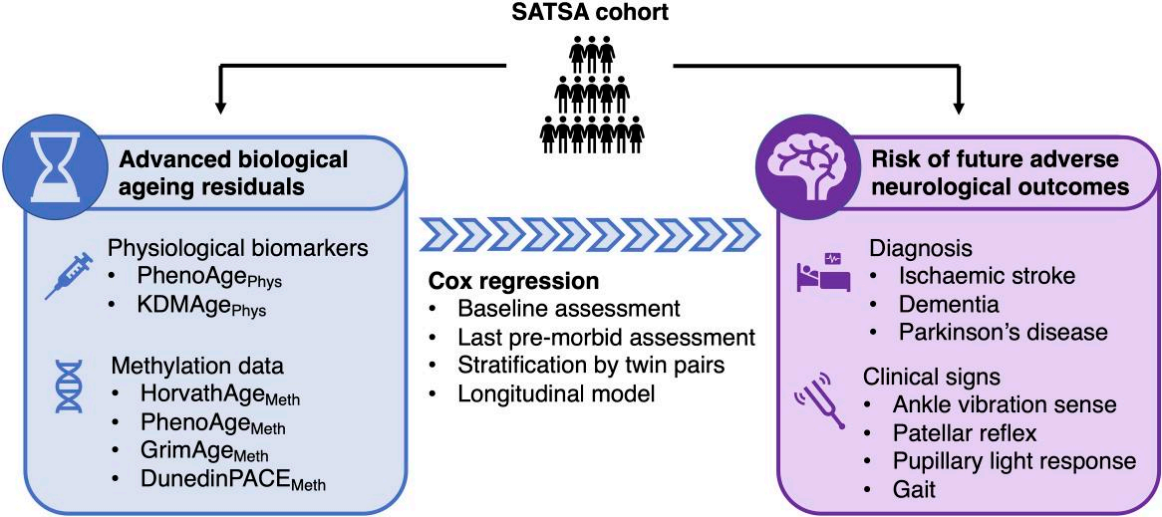
**Schematic representation of the study.** Measures of biological age (BA) were calculated for participants of the SATSA cohort study, and biological ageing residuals determined by regression on chronological age. Cox survival models were then used to determine whether advanced biological age residuals were associated with subsequent risk for developing a neurological condition or developing abnormal features on neurological examination. CA = chronological age; SATSA = Swedish Adoption/Twin Study of Aging.

The Swedish Adoption/Twin Study of Aging (SATSA) is a cohort study encompassing longitudinal data on 857 individuals collected in-person from 1984 to 2014.^26^ This includes clinical assessments, basic laboratory blood tests and leucocyte DNA methylation data, with information available on diagnoses followed-up over several decades. The use of a twin cohort additionally allows for some adjustment of unobserved cofounding factors, such as any genetic predisposition to certain disorders. The present study generates measures of biological age based on clinical biomarkers or methylation data and tests the hypothesis that higher biological age measures than expected for chronological age can predict future risk of age-associated neurological diagnoses or development of abnormal neurological signs (Fig. 1).

## Materials and methods

### Study population

SATSA is a Swedish cohort study that began in 1984, based on all same-sex twin pairs in the Swedish Twin Registry born before 1959 who were identified as reared apart matched to twin pairs reared together based on sex, date and county of birth (*n* = 3838 individuals; mean year of birth = 1919; 57.9% female).^26^ The SATSA data collections started with a questionnaire sent out to 2845 individuals in 1984, to which 2018 responded. Twin pairs over the age of 50 who both answered the first questionnaire were invited to in-person assessments, including health examinations, neurological tests and collection of blood samples. In total, *n* = 857 individuals participated in at least one of the nine in-person assessments, carried out between 1985 and 2014 (mean year of birth = 1925; 59.6% female). The details of in-person assessments and blood sampling have been previously described by Pedersen *et al*.^27^ The study has approval from the Swedish Ethical Review Board in Stockholm.

### Markers of biological ageing

Markers of biological age (Box 1) were generated for every available time point for all individuals within SATSA. Clinical biomarker data were available from 3175 instances across 802 individuals, which were used for the generation of two ‘physiological ages’: PhenoAge_Phys_ and KDMAge_Phys_. DNA methylation data were available from 900 instances in 365 individuals and was used to generate three ‘methylation ages’: HorvathAge_Meth_, PhenoAge_Meth_ and GrimAge_Meth_ as well as the DunedinPACE_Meth_ measure of the rate of ageing. Biological age residuals were then generated by regressing each biological age measure on chronological age, such that a biological age residual of +1.0 suggests an individual's biological age measure is 1 year older than expected for their chronological age. Residuals were then divided by their respective standard deviation (SD) so that the effect sizes reported can be compared between different biological age measures.

Box 1 **Biological ageing markers used in the present study**

**Physiological ageing markers**

**PhenoAge_Phys_**: Uses clinical biomarker results and chronological age to estimate the age at which the participant's mortality risk would be expected.^5,28^
**KDMAge_Phys_**: Uses clinical biomarker results and chronological age to estimate a latent variable (biological age) by the Klemera-Doubal method.^8,28^

**Methylation ageing markers**

**HorvathAge_Meth_**: Uses principal components from methylation data to estimate the participant's chronological age.^7,29^
**PhenoAge_Meth_**: Uses principal components from methylation data to estimate the age at which the participant's mortality risk would be expected, based on the original panel of PhenoAge biomarkers.^5,29^
**GrimAge_Meth_**: Uses principal components from methylation data to estimate the age at which the participant's mortality risk would be expected, based on methylation surrogates for seven plasma proteins and smoking status.^6,29^
**DunedinPACE_Meth_**: Uses methylation data from a single time point to estimate the preceding rate of change of a panel of physiological biomarkers.^30^

#### Physiological ages

**Figure 2:**
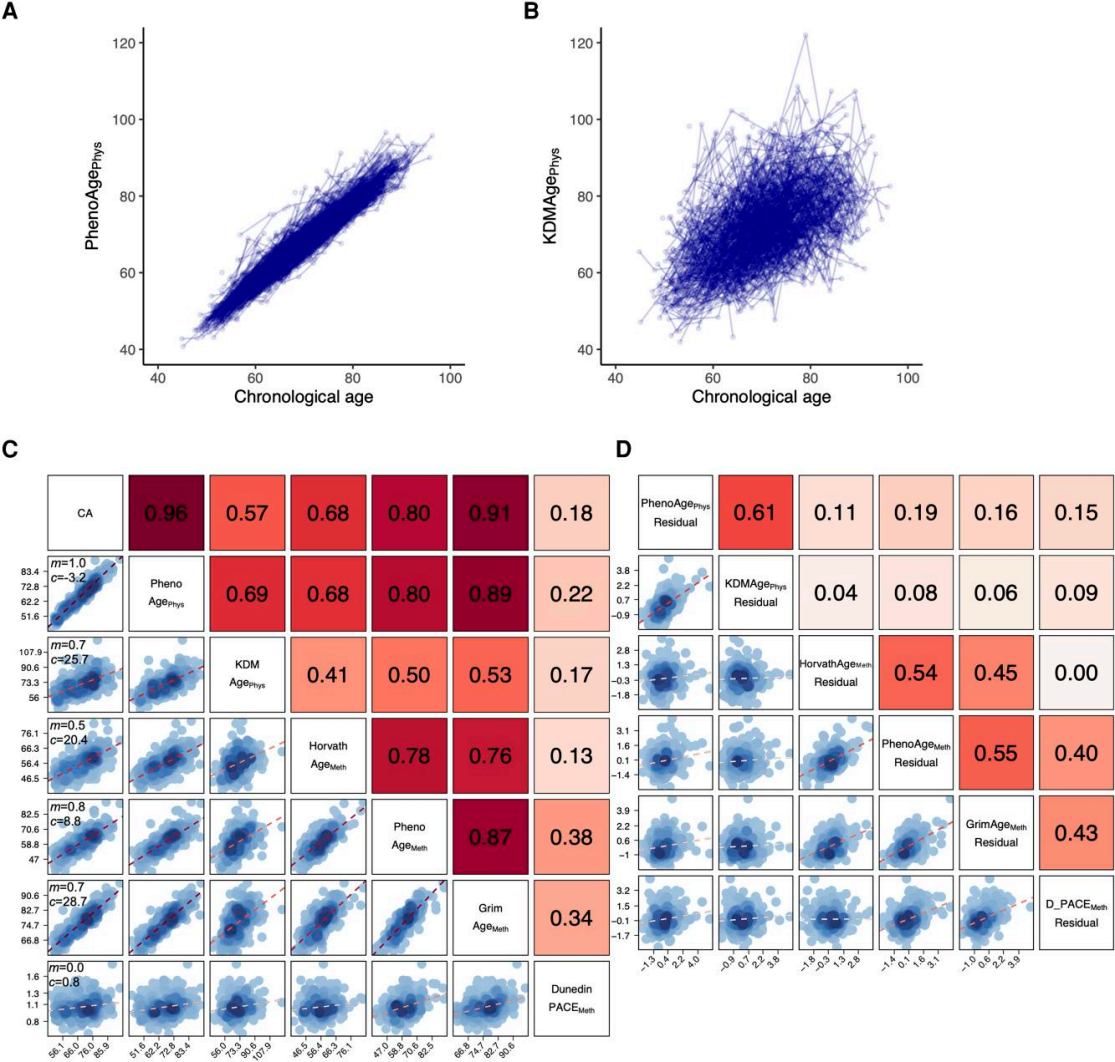
**Visualization of the different biological age measures amongst SATSA participants.** PhenoAge_Phys_ (**A**) and KDMAge_Phys_ (**B**) are shown for all assessments of all participants plotted against chronological age (CA). Each point represents an individual assessment, with longitudinal assessments from the same individual joined by a line. Correlation matrices for baseline assessments of the six biological age measures and chronological age (**C**) and the six biological age measure residuals (**D**) are shown, with scatter plots in the *bottom left* and Pearson correlation coefficients (*r*) in the *top right*. In **C**, in the left-hand column the gradient (*m*) and intercept (*c*) are displayed for the linear regression of each biological age measure on chronological age.

Two physiological age measures (PhenoAge_Phys_ and KDMAge_Phys_) were generated using the *BioAge* package in *R*, which trains models based on shared biomarkers measured in the US Health and Nutrition Examination Surveys (NHANES).^28^ Clinical biomarkers measured both in NHANES III and longitudinally in SATSA (Supplementary Fig. 1) were selected if they correlated with chronological age (|*r*| > 0.1) amongst NHANES III participants (Table 1). PhenoAge_Phys_—based on phenotypic age but using our available panel of biomarkers—is trained to predict the age at which the same mortality would be expected given this panel of biomarker results.^5^ KDMAge_Phys_—created using the Klemera-Doubal method—is trained to estimate a latent variable (biological age) given chronological age and the panel of biomarkers, with parallel analysis for males and females.^8^ Both measures were validated in the NHANES IV testing set using methods previously described and showed statistically significant associations with all-cause mortality adjusted for chronological age.^28^ In SATSA, both measures correlated highly with chronological age and other measures of biological age (Fig. 2).

**Table 1:** Correlation between available clinical biomarkers and chronological age in NHANES III

	Pearson correlation coefficient (*r*)		
	All	Males	Females
(BMI)	(−0.02)	(0.01)	(0.03)
(Weight)	(−0.06)	(−0.07)	(−0.07)
**Waist circumference**	**0.26**	**0.31**	**0.23**
**Systolic blood pressure**	**0.59**	**0.51**	**0.66**
**Diastolic blood pressure**	**0.11**	(0.06)	**0.16**
**Pulse rate**	**0.61**	**0.54**	**0.67**
**Glucose**	**0.26**	**0.24**	**0.28**
**Total cholesterol**	**0.32**	**0.19**	**0.42**
**Triglycerides**	**0.18**	**0.10**	**0.26**

Coefficients shown in bold without parentheses are those biomarkers with |*r*| > 0.1, which were used for training the physiological age models. BMI = body mass index.

#### Methylation ages

DNA methylation from blood leucocytes was assayed using the Illumina Infinium Human Methylation 450K BeadChip according to the manufacturer's instructions.^31^ Three measures of methylation age (HorvathAge_Meth_, PhenoAge_Meth_ and GrimAge_Meth_) were generated using principal components of CpG-level data as input to maximize their reliability, using the method described by Higgins-Chen *et al*.^29^ The DunedinPACE_Meth_ measure was generated using the method described by Belsky *et al*.^30^

#### Residuals of biological ageing

Biological age residuals were calculated from the regression of each biological age assessment on chronological age, modelled as a natural spline with three degrees of freedom:


(1) $$BA=ns(CA,df=3)+BA_{Residual}$$

No adjustments for covariates, relatedness between twin pairs or repeated measurements from the same individual were made to calculate the biological age residuals, as these were subsequently adjusted for downstream, when analysing the effect of these residuals on the outcome measures.

### Outcome measures

Information on diagnoses was obtained from the Swedish National Patient Register and the Causes of Death Register, updated to the end of 2016. ICD codes were used to identify cases of ischaemic stroke (I63), dementia (F00-F03, F05.1, G30, G31.1, G31.8A) and Parkinson's disease (G20). Two additional sources were available to detect cases of dementia: first from clinical work-up within SATSA and second, the presence of dementia medications in the Prescribed Drug Register, as previously described.^32^ Follow-up ended when the participant died or was otherwise censored on 31 December 2016. Mean follow-up time for neurological diagnoses was 18.4 years from baseline assessment (mean chronological age at last follow-up = 82.8 years).

Neurological examinations were performed by trained nurses at a location convenient to the participant's home. Ankle vibration sense, patellar reflex, direct pupillary response to light and gait were extracted from a wider neurological examination due to their known associations with chronological age^21-24^ and inclusion within all waves one to eight of SATSA. For ankle vibration sense, participants were asked if they could feel a tuning fork vibrating against the bone of the ankle on either foot (‘yes’, ‘no’ or ‘inbetween’). For patellar reflexes, the nurse recorded whether they observed normal knee extension in response to striking the patellar tendon with a tendon hammer (‘yes’, ‘no’ or ‘inbetween’). For direct pupillary response to light, the nurse recorded whether they observed pupillary constriction when a flashlight was moved over the ipsilateral eye from the side (‘yes’, ‘no’ or ‘inbetween’). For gait, the nurse recorded whether a participant was able to walk for a distance of 30 m (‘yes’, ‘with difficulty’ or ‘impossible’). In the present analysis, an unremarkable examination (‘yes’) bilaterally was considered as ‘normal’, whilst any type of deficit on either side was considered ‘abnormal’. Owing to some variability between normal and abnormal assessments longitudinally, abnormal assessments were discounted if they were subsequently followed by two or more normal assessments (Supplementary Fig. 2). For survival analysis, time to first abnormal assessment was then considered, with participants censored at the time of their last normal examination. Mean follow-up time for neurological examinations was 11.5 years from baseline assessment (mean chronological age at last follow-up = 76.0 years).

### Statistical methods

#### Cox regression analysis

Cox proportional hazards regression was used to assess the effect of a 1 SD increase in biological age residual on the hazard of each of the outcomes described, using the *survival* package in *R*. Either the baseline or the latest premorbid assessment of biological age residual was used in a ‘single assessment’ model. Baseline assessments were the earliest recorded for each individual in SATSA, excluding individuals who were diagnosed prior to their baseline testing. Latest premorbid assessments were the last assessments recorded prior to diagnosis for cases, or prior to reaching the median age of diagnosis for non-cases.

As not all participants with physiological ages (*n* = 802) available had methylation ages (*n* = 365), two separate analyses were performed: first using the largest sample available for each biological age marker, and second, using the 365 individuals with complete data across all six markers of biological age. The former analysis (with the largest available samples) is shown in the main figures with the sample sizes displayed in the legends. The latter (with a smaller but consistent sample for all markers of biological age) is included in the Supplementary material. Associations with biological age residual were adjusted for sex, body mass index (BMI), smoking status (ever versus never smoker) and education level (primary versus above primary level). For the neurological examination outcomes, models were further adjusted for the presence of an ischaemic stroke, dementia or Parkinson's disease diagnosis. Where indicated, a rate of change of biological age measure between the baseline and latest premorbid assessment (the difference between biological age measured at the two assessments divided by the time between these) was additionally incorporated into the model. Assessments with missing data in any relevant field were dropped from analysis. Chronological age was implicitly adjusted for by using attained age as the underlying timescale. All models were stratified by decade of birth and robust standard errors were used to account for relatedness within a twin pair. Proportional hazard assumptions were tested for each model using the Schoenfeld residual test.

To adjust for other, unmeasured, factors (such as genetics) where indicated, survival models were also stratified by twin pair. Monozygotic and dizygotic twin pairs were handled in the same way.

#### Longitudinal analysis

To investigate whether chronological age modulates the association between advanced biological age and neurological outcomes, a time-dependent coefficient model was generated incorporating all assessments of biological age for each individual prior to the occurrence of the respective outcome. For each outcome, data were split into five chronological age intervals (<60, 60–70, 70–80, 80–90 and >90) and only intervals with ≥10 observed cases were used for further analysis to minimize skew from small numbers of outlier values. The proportional hazards models described in the ‘Cox regression analysis’ section were expanded to include an interaction between chronological age interval and biological age residual in this dataset. Adjustment for covariates, decade of birth and relatedness within twin pairs were handled in the same way. Where the proportional hazards assumption was not met for another covariate, results were similarly verified by allowing this covariate to vary with chronological age interval.

*P*-values <0.05 were interpreted as statistically significant. Adjustment was not made for multiple comparisons across the six biological age measures, given these are correlated and not independent (Fig. 1), but *P-*values and 95% confidence intervals (CI) are reported for each analysis. All analyses were conducted using R version 4.0.5.

## Results

### Biological ageing residuals and risk of neurological diagnoses

Baseline characteristics of the population including the six different biological age measures are shown in Table 2. Amongst follow-up of the 802 SATSA participants with biological age assessments, there were 179 individuals diagnosed with dementia, 116 with ischaemic stroke and 15 with Parkinson's disease. These three age-related neurological disorders were prioritized for further analysis, whilst other neurological conditions (including multiple sclerosis, motor neuron disease and epilepsy) had too few cases to offer adequate statistical power.

**Table 2:** Baseline characteristics of the population under study

	Full population (*n* = 802)	Subpopulation with methylation data (*n* = 365)
**Chronological age, years**	64.5 (8.9)	68.7 (9.4)
**BA marker, years**		
PhenoAge_Phys_	62.5 (9.7)	66.7 (10.0)
KDMAge_Phys_	70.7 (11.4)	71.3 (11.0)
HorvathAge_Meth_	–	56.3 (7.2)
PhenoAge_Meth_	–	60.4 (8.8)
GrimAge_Meth_	–	76.2 (7.2)
DunedinPACE_Meth_^a^	–	1.1 (0.2)
**BA marker residual, years**		
PhenoAge_Phys_ residual	0.1 (2.7)	−0.2 (2.9)
KDMAge_Phys_ residual	2.5 (8.8)	0.3 (9.1)
HorvathAge_Meth_ residual	–	−0.6 (5.3)
PhenoAge_Meth_ residual	–	0.1 (5.2)
GrimAge_Meth_ residual	–	−0.2 (3.0)
DunedinPACE_Meth_ residual^a^	–	0.0 (0.2)
**Model covariates**		
Female sex: *n* (%)	472 (58.9%)	215 (58.9%)
BMI: kg/m^2^	25.7 (4.1)	26.3 (4.1)
Current or ex-smoker: *n* (%)	278 (35.7%)	70 (21.2%)
Above primary education: *n* (%)	310 (39.9%)	160 (45.2%)

Data for the full population and the subpopulation with methylation data available are shown separately for the baseline assessment. Values are displayed as mean (standard deviation, SD) unless otherwise indicated. BA = biological age; BMI = body mass index.

^a^DunedinPACE is reported in units of years of physiological decline per one chronological year; all other biological age markers are reported in units of years.

#### Baseline assessments of biological age

**Figure 3:**
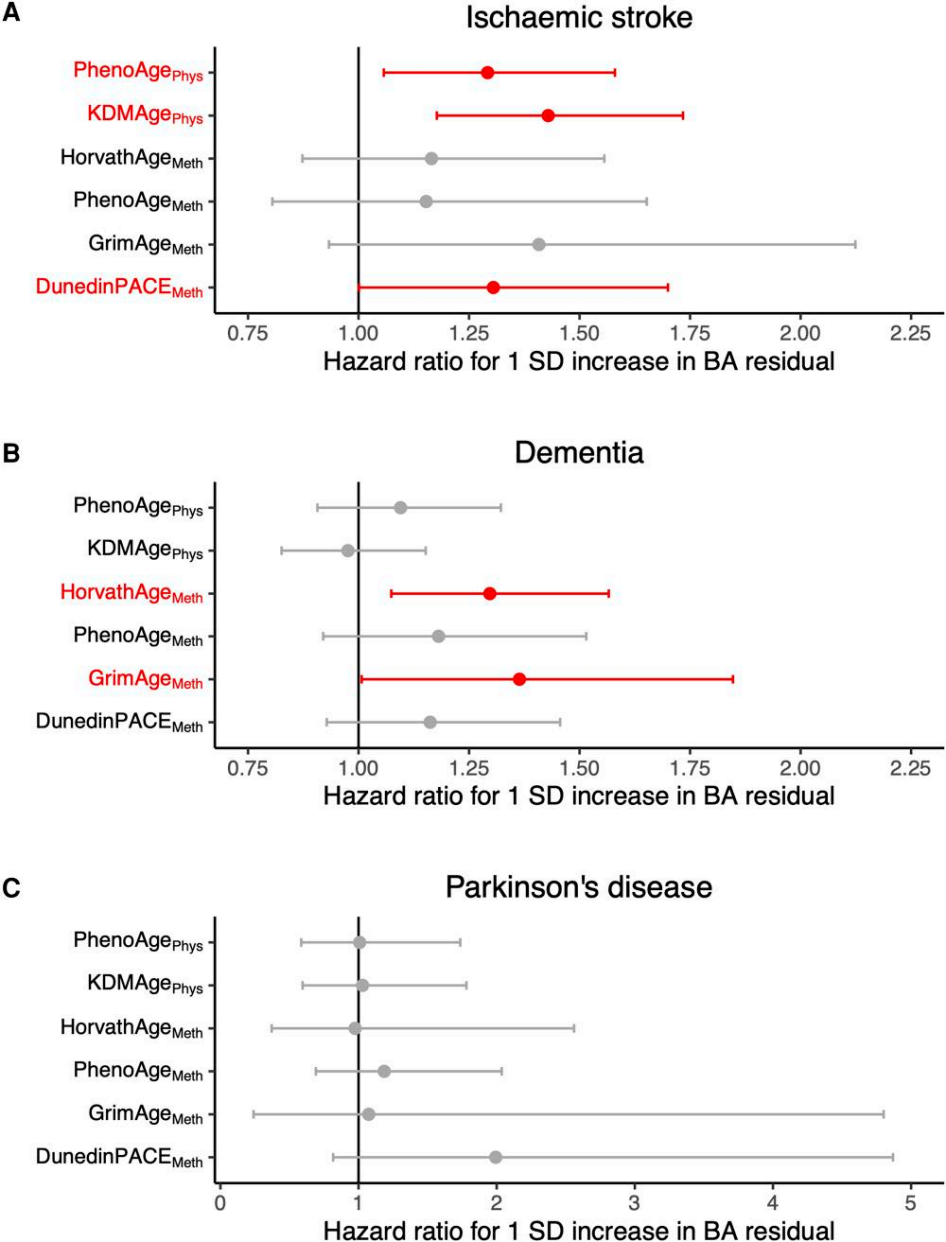
**Effect of baseline biological age residual on risk of subsequent neurological diagnosis.** The hazard ratios and 95% confidence intervals (CIs) of a 1 standard deviation (SD) increase in each biological age (BA) residual are shown for ischaemic stroke (**A**), dementia (**B**) and Parkinson's disease (**C**). For PhenoAge_Phys_ and KDMAge_Phys_, *n_total_* = 793, 791 and 800 for ischaemic stroke, dementia and Parkinson's disease analyses, respectively. For HorvathAge_Meth_, PhenoAge_Meth_, GrimAge_Meth_ and DunedinPACE_Meth_, *n_total_* = 358, 355 and 365 for ischaemic stroke, dementia and Parkinson's disease analyses, respectively. *n_total_* = total population at risk at the time of the baseline assessment.

To study the effect of increasing biological age residual on the risk of being diagnosed with an age-related neurological disorder, we used Cox regression models with participants’ baseline assessment for each biological age measure (Fig. 3 and Supplementary Tables 1 and 2). A 1 SD increase in either PhenoAge_Phys_ or KDMAge_Phys_ residual significantly increased the risk of ischaemic stroke diagnosis by 29.2% [hazard ratio (HR): 1.29, 95% CI 1.06–1.58, *P* = 0.012] and 42.9% (HR 1.43, CI 1.18–1.73, *P* = 3.1 × 10^−4^), respectively (Fig. 3A). A 1 SD increase in the four methylation biological age measures all contributed to a higher risk of ischaemic stroke, which was significant only for DunedinPACE_Meth_ (HR 1.30, CI 1.00–1.70, *P* = 0.049), albeit analysed in a smaller population than for the physiological biological age measures. In contrast, for dementia risk (Fig. 3B), neither physiological biological age measure had a statistically significant effect, but two methylation biological age measures significantly increased the risk: HorvathAge_Meth_ (HR 1.30, CI 1.07–1.57, *P* = 0.007) and GrimAge_Meth_ (HR 1.36, CI 1.01–1.85, *P* = 0.045). No biological age residual at baseline had an effect on the risk of Parkinson's disease diagnosis.

#### Latest premorbid assessments of biological age

To compare these baseline results to a biological age assessment close to the time of diagnosis, we next considered the latest premorbid assessment of each biological age marker on disease risk (Supplementary Tables 3 and 4). For the physiological biological age measures (PhenoAge_Phys_ and KDMAge_Phys_) the latest premorbid assessments were carried out a median of 10.7, 11.6 and 9.6 years after the baseline assessments for ischaemic stroke, dementia and Parkinson's disease, respectively. For the methylation biological age measures (HorvathAge_Meth_, PhenoAge_Meth_, GrimAge_Meth_ and DunedinPACE_Meth_) the corresponding intervals between baseline and latest premorbid assessments were 5.1, 6.5 and 3.3 years, respectively.

Similar to the baseline assessments, a 1 SD increase in either physiological biological age residual at the latest premorbid assessment increased the risk of future ischaemic stroke diagnosis, with an effect size of 31.8% for PhenoAge_Phys_ residual (HR 1.32, CI 1.10–1.57, *P* = 0.002) and 26.9% for KDMAge_Phys_ residual (HR 1.27, CI 1.05–1.53, *P* = 0.013). DunedinPACE_Meth_ had a similar effect size on ischaemic stroke when measured at the latest premorbid assessment compared to baseline (HR 1.34, CI 1.01–1.78, *P* = 0.042). Once again, the risk of dementia diagnosis was more dependent upon methylation biological age residuals, with statistically significant effects of a 1 SD increase in HorvathAge_Meth_ (HR 1.27, 1.03–1.57, *P* = 0.027) and PhenoAge_Meth_ (HR 1.28, 1.00–1.64, *P* = 0.048). Effect sizes were generally similar to those seen at baseline. A higher DunedinPACE_Meth_ at this later time point also significantly increased the risk of developing Parkinson's disease (HR 3.44, CI 1.07–11.05, *P* = 0.038).

At the time of the latest premorbid assessment, we also have access to a ‘rate of change’ of biological age for individuals that participated in multiple biological age assessments prior to diagnosis. After adjustment for the latest premorbid biological age, a 1 SD increase in the rate of change in biological age was not significantly associated with the future risk of any of the diagnoses studied, with effect sizes tending to be >1.0 for dementia and Parkinson's disease and <1.0 for ischaemic stroke (Supplementary Tables 5 and 6).

#### Stratification by twin pair

To provide some adjustment for unmeasured confounding factors, we next leveraged the twin structure of SATSA to stratify survival models by twin pairs. The trade-off for this was a smaller sample size, given that pairs in which neither twin developed the relevant outcome could not contribute to the stratified analysis. Amongst the 802 individuals with at least one biological age assessment, there were 31 concordant and 102 discordant twin pairs with dementia, 15 concordant and 80 discordant pairs with stroke and 13 twin pairs with Parkinson's disease (all discordant). Effect sizes for baseline physiological age residuals on ischaemic stroke risk were similar when stratified by twin pair: 58.9% for PhenoAge_Phys_ residual (HR 1.59, CI 0.93–2.71, *P* = 0.089) and 96.8% for KDMAge_Phys_ residual (HR 1.97, CI 1.18–3.27, *P* = 0.009). In the subgroup with methylation data available, effect sizes were positive for most methylation age residuals on both ischaemic stroke and dementia risk, but none were statistically significant (Supplementary Tables 7 and 8).

#### Chronological age-dependent effects

**Figure 4:**
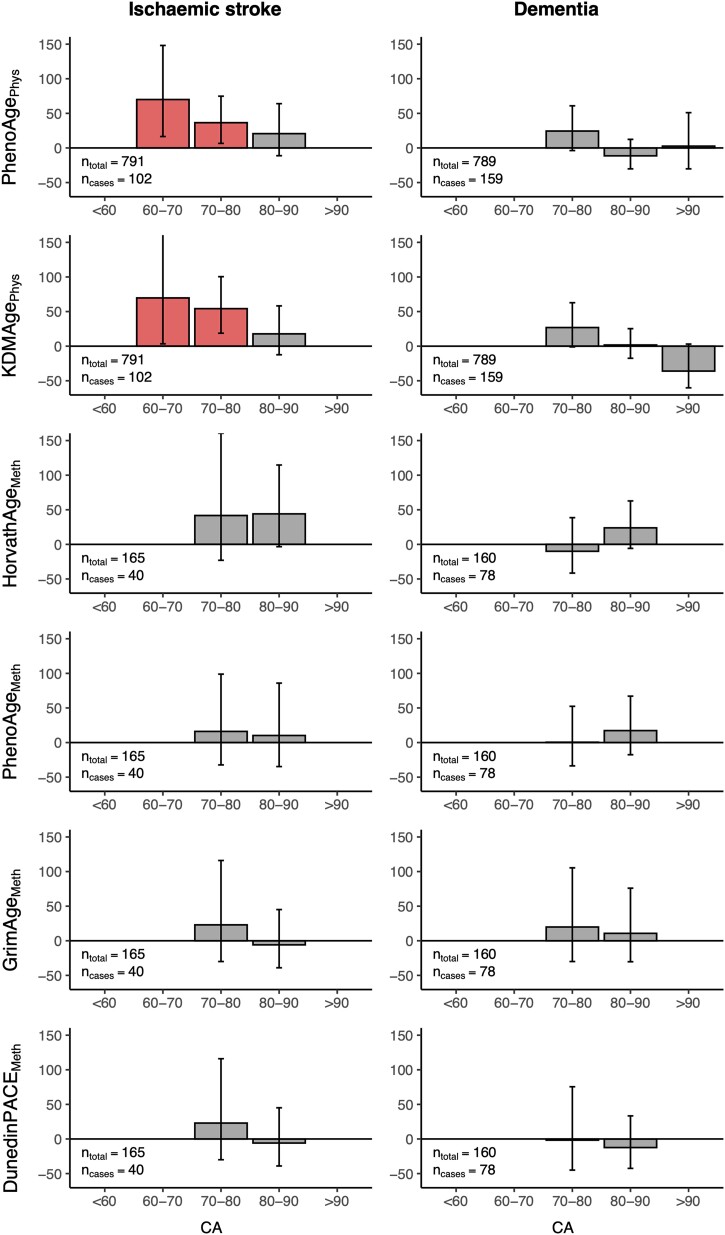
**Effect of biological age residual on risk of subsequent neurological diagnosis at different chronological ages.** The percentage increases in hazard associated with a 1 standard deviation (SD) increase in each biological age (BA) residual are shown, split by chronological age group for ischaemic stroke and dementia. The numbers of individuals in each analysis are reported on the plots. Bars are coloured red for a statistically significant effect and grey for a non-significant effect on hazard. *n_total_* = total population at risk; *n_cases_* = number of cases during follow-up.

For some of the above models, there was evidence that the effect of biological age residual was not constant across chronological age (Schonfeld residual test *P* < 0.05). Many participants had repeated biological age assessments over time prior to their diagnosis, which allowed us to interrogate how the effect of accelerated biological age on the risk of neurological diagnosis varied with increasing chronological age. To explore this, we produced a model using premorbid longitudinal data in which the coefficient for biological age could vary by decade of chronological age (<60, 60–70, 70–80, 80–90 and >90) (Fig. 4).

The association between advanced physiological biological age and ischaemic stroke risk was most pronounced at younger chronological age, diminishing above a chronological age of 80. The same chronological age-dependent trend was not observed for dementia, or for the methylation ages in ischaemic stroke or dementia. A similar analysis for Parkinson's disease was limited by small numbers of cases within each chronological age category.

To summarize, a premature acceleration in two physiological markers of biological age generated from basic clinical biomarkers was robustly associated with an increased risk of future ischaemic stroke. This association diminished with increasing chronological age. Meanwhile, acceleration in measures of epigenetic biological age were associated with increased risk of future dementia diagnosis.

### Biological ageing residuals and the risk of abnormal neurological examination findings

As it is also common to have some degree of age-related neurological dysfunction outside of specific diagnoses, we next explored the effect of biological age on neurological examinations carried out longitudinally on participants both with and without diagnosed neurological conditions.

Of the participants with at least one biological age assessment, all 802 (100%) had a recorded neurological examination, with a median of 4.0 longitudinal examinations per participant. Four neurological assessments were chosen for analysis based on known associations with advancing chronological age^21–24^ and the availability of longitudinal data in SATSA: ankle vibration sense, patellar reflex, direct pupillary response to light and assessment of gait (Supplementary Fig. 2). The lifetime prevalence of abnormal signs in each of these assessments in the study population is shown in Supplementary Table 9.

#### Baseline assessments of biological age

**Figure 5:**
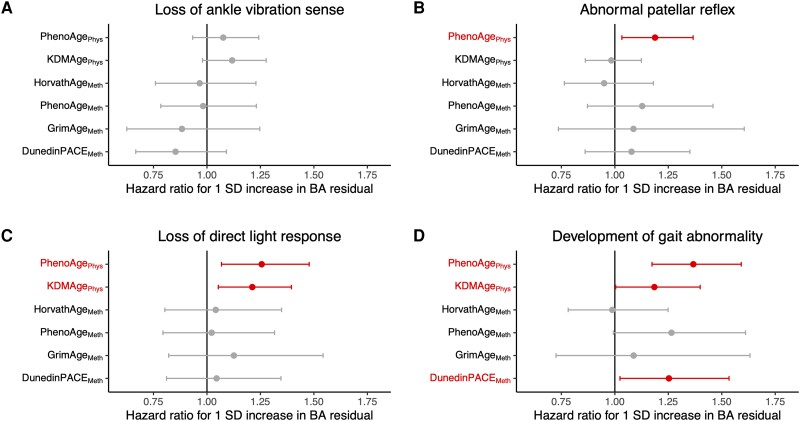
**Effect of baseline biological age residuals on risk of development of abnormal clinical signs on neurological examination.** The hazard ratios and 95% CIs of a 1 standard deviation (SD) increase in each biological age residual are shown for loss of vibration sense at the ankle (**A**), development of an abnormal patellar tendon reflex (**B**), loss of a normal direct pupillary reflex response to light (**C**) and development of a gait abnormality (**D**). For PhenoAge_Phys_ and KDMAge_Phys_, *n_total_* = 577, 628, 673 and 658 for ankle vibration, patellar reflex, pupillary light reflex and gait analyses, respectively. For HorvathAge_Meth_, PhenoAge_Meth_, GrimAge_Meth_ and DunedinPACE_Meth_, *n_total_* = 222, 234, 273 and 269 for ankle vibration, patellar reflex, pupillary light reflex and gait analyses, respectively. *n_total_* = total population at risk at the time of the baseline assessment.

At the baseline assessment, accelerated physiological biological age measures tended to increase the risk of all abnormal signs (Fig. 5 and Supplementary Tables 10 and 11), with statistically significant effects of PhenoAge_Phys_ residual on patellar reflex (HR 1.19, CI 1.03–1.37, *P* = 0.016), pupillary light response (HR 1.26, CI 1.07–1.48, *P* = 0.006) and abnormal gait (HR 1.37, CI 1.17–1.59, *P* = 5.5 × 10^−5^) and of KDMAge_Phys_ on pupillary light response (HR 1.21, CI 1.05–1.40, *P* = 0.007) and gait (HR 1.18, CI 1.00–1.40, *P* = 0.045). These results were consistent regardless of adjustment for the presence or absence of the age-related neurological diagnoses explored previously (ischaemic stroke, dementia and Parkinson's disease). Of the baseline epigenetic biological age residuals at baseline, only DunedinPACE_Meth_ contributed a significant effect on the development of gait abnormalities (HR 1.25, CI 1.02–1.53, *P* = 0.028).

#### Latest premorbid assessments of biological age

Similarly, when using the latest premorbid assessments, a 1 SD increase in residual for the two physiological ages tended to increase the risk of abnormal neurological signs, with a statistically significant effect of increased PhenoAge_Phys_ residual on the risk of losing ankle vibration sense (HR 1.15, CI 1.00–1.32, *P* = 0.048) or developing an abnormal patellar reflex (HR 1.17, CI 1.06–1.29, *P* = 0.002). Epigenetic biological age markers measured premorbidly tended to have positive associations with future neurological signs, and significant effects were detected for abnormal gait with PhenoAge_Meth_ (HR 1.24 CI 1.03–1.49, *P* = 0.020) and GrimAge_Meth_ (HR 1.31, CI 1.01–1.70, *P* = 0.038) (Supplementary Tables 12 and 13). We further studied whether the rate of change in biological age residual from baseline to latest premorbid assessment contributed to the risk of incident abnormal neurological signs (Supplementary Tables 14 and 15). In this population, a higher rate of change in biological age did not significantly increase the future risk of neurological signs for any of the biological age measures assessed. There was a negative association between rate of change of PhenoAge_Phys_ and future development of gait abnormality (HR 0.79, CI 0.67–0.92, *P* = 0.002), consistent with a higher effect size of PhenoAge_Phys_ on this outcome at the baseline compared to the latest premorbid assessment.

#### Stratification by twin pair

Equivalent survival analysis was next performed with stratification by twin pair. Amongst the 802 participants, there were 151 concordant and 168 discordant twin pairs with abnormal ankle vibration sense, 107 concordant and 156 discordant pairs with abnormal patellar reflexes, 55 concordant and 144 discordant pairs with abnormal pupillary light responses and 52 concordant and 154 discordant for gait abnormalities. When stratified by twin pair, no baseline biological age residuals contributed a significant effect on development of neurological signs (Supplementary Tables 16 and 17).

#### Chronological age-dependent effects

Finally, the effects of increased biological age residuals on neurological examination findings were explored in a model incorporating longitudinal premorbid assessments, in which the hazard ratio could vary with chronological age (Supplementary Fig. 3). Advanced physiological biological age measures were associated with loss of ankle vibration sense for individuals in their seventh decade, whilst both physiological biological age measures and PhenoAge_Meth_ in the eighth decade of life predicted future gait abnormalities. Similarly to what was seen with neurological diagnoses (Fig. 4), the effect sizes were generally small above a chronological age of 80, beyond which there were no statistically significant associations between advanced biological age and development of abnormal neurological signs.

In summary, accelerated biological age residuals generally tended to increase the risk of developing age-related neurological examination findings, independent of three common age-related neurological diagnoses. The strongest evidence for this was for associations between accelerated physiological biological age measures and future risk of abnormal patellar reflex, pupillary response and gait, albeit with some variability between different types of analysis.

## Discussion

In this study, we explored the effect of advanced biological age on the future risk of developing age-related neurological conditions or clinical signs. We used data from SATSA, a cohort study in which twin pairs had multiple assessments of biological age both before and after these outcomes.

Of the three age-associated neurological disorders studied, an increase in biological age residual was most robustly associated to ischaemic stroke risk. For the two physiological biological age measures (based on clinical biomarkers), significant effects were seen from both baseline and latest premorbid assessments and then verified by stratifying the analysis by twin pairs. Across these different approaches, the effect size seen was ∼30–60% increased risk of ischaemic stroke for every SD increase in PhenoAge_Phys_ residual, and 25–95% increased risk for equivalent increments in KDMAge_Phys_ residual. For reference, 1 SD increase corresponds to 2.7 years of advancement in PhenoAge_Phys_, 8.8 years of advancement in KDMAge_Phys_ and 2.9–5.3 years of advancement in the methylation biological age measures (Table 2). The strength of these associations for ischaemic stroke presumably reflects the reliance of these physiological biological age measures on cardiometabolic health: the biomarkers used included waist circumference, blood pressure, glucose and lipids, all of which are well established risk factors for ischaemic stroke.^2^ The methylation biological age markers (which are less intimately related to cardiometabolic risk factors), also had similar effect sizes, with estimates ranging from 15–67%. However, these effects were generally not statistically significant, likely in part due to fewer measurements of epigenetic ages.

Unlike for ischaemic stroke, physiological biological age acceleration was a weak predictor of dementia risk. Whilst there is evidence that dementia risk is influenced by cardiometabolic risk factors,^33^ this is less strong than the association for ischaemic stroke (a CNS manifestation of systemic cardiovascular disease), consistent with our results. Conversely, three methylation clocks (HorvathAge_Meth_, PhenoAge_Meth_ and GrimAge_Meth_) all had positive associations with future dementia diagnosis, with effect size estimates ranging from 11–212%. There are mixed reports in the literature, with some studies demonstrating a similar positive association between blood epigenetic age and dementia risk,^13,14^ and others reporting no effect.^34,35^ A study in the Framingham Heart Study Offspring cohort found that DunedinPACE and HorvathAge were more strongly associated with future dementia risk than PhenoAge and GrimAge.^14^ We report here similar effect sizes in SATSA using the updated principal component-based versions of the methylation clocks, but also found that the increased dementia risk from advanced methylation biological age was no longer statistically significant when stratifying by twin pairs. It is difficult to say whether this was due to unmeasured confounders biasing the unstratified analysis, or simply a reflection of the smaller sample size once limited to dementia-affected twin pairs.

The other part of our analysis focused on the detection of age-associated clinical deficits on neurological examination. Across different models, a 1 SD increase in physiological biological age measures was generally associated with increased future risk of losing normal ankle vibration sense (estimate range −2 to 15%), patellar tendon reflexes (−2 to 19%), direct pupillary response to light (2 to 35%) or gait (−16 to 37%). Meanwhile, epigenetic biological age measures had some value in predicting abnormal gait or pupillary response, particularly when measured at the latest premorbid assessment.

The four neurological assessments explored here all represent combined functions of the CNS and peripheral nervous system (PNS). Vibration sense relies on intact sensory neurons and the dorsal column medial lemniscus pathway, the patellar reflex on peripheral afferent and efferent neurons via the spinal cord and pupillary response on the functions of cranial nerves II and III with relevant brainstem circuits. Gait, meanwhile, relies on a complex interaction between sensory inputs, motor outputs and central processing. Either neurology-specific or more systemic age-related pathology could therefore contribute to the development of any of these abnormalities; e.g. musculoskeletal problems influencing gait or ocular pathology affecting pupillary response. Cognitive assessments, which are more specific to CNS function, have been previously described for SATSA and show correlation with other biological age residuals over time.^9^ Our analysis was agnostic to the underlying pathology, hence this heterogeneity may contribute to the variable effects on biological age-predicted hazards that we observed.

A strength of this study is the access to repeated samples prior to relevant outcomes occurring, revealing more information about the temporal relationship between biological age and neurological disease. For example, advanced neurological conditions will cause changes in diet, activity, peripheral immunity and risk of intercurrent infections, which could influence biomarkers used to calculate biological age. We found here that acceleration in biological age predates stroke or dementia diagnosis by several years, which is encouraging for the clinical utility of biological age measures for identifying people at risk with sufficient time for a putative intervention. A caveat of our approach is that the diseases studies here all have subclinical prodromes that can predate clinical diagnosis considerably: e.g. cerebrovascular atherosclerosis in stroke, or accumulation of amyloid plaque in Alzheimer's disease dementia. Consequently, there remains a risk of reverse-causation; the ‘latest premorbid’ assessments in particular are likely to occur during this prodromal period. We have tried to mitigate for this by reporting the effects of biological age both at baseline and at latest premorbid assessments.

The repeated samples also allow us to assess changes over time in the biological age measures, and whether this has additional utility in predicting neurological outcomes. In general, we did not find any evidence that a more rapidly increasing biological age was associated with a higher risk of any neurological outcome. In fact, some outcomes (particularly ischaemic stroke and gait abnormalities) tended to be negatively associated with rate of change, after adjustment for the latest premorbid value. Similar conclusions were reached from a study of an overlapping cohort, in which the rate of change of the frailty index had no effect on mortality.^36^ In contrast, other work has demonstrated that the pace of ageing in a younger cohort predicts age-associated neurological outcomes including cognitive function and MRI-based brain age.^37,38^ One synthesis of these findings is that rates of change in biological age measures might be more critical earlier in life, whereas for older populations such as SATSA (mean age 64.5 at baseline assessment) a sustained biological age advancement over time is more detrimental for age-associated morbidity and mortality. That said, DunedinPACE_Meth_, which is a methylation-based predictor for the pace of ageing, increased risk for both stroke and gait abnormalities in our cohort, although the latter effect was attenuated at the latest premorbid time point.

The measures of biological age analysed here all focus on peripheral biomarkers, such as serum chemistry and leucocyte epigenetic markers. Previous work has demonstrated how these can be incongruent with measures of brain biological age from the same individuals.^39^ Stronger associations may be observed by incorporating CNS biomarkers such as neurofilament light chain.^20^ However, an advantage of focusing on well established peripheral biomarkers is the ability to use biological age measurements dating back to the 1980s, with several decades of follow-up data.

Finally, for all positive associations identified between advanced biological age and neurological outcomes, we found that the effect sizes were generally small above a chronological age of 80. Similar to previous work looking at all-cause mortality,^40^ it appears that advanced biological age has a lower positive predictive value for neurological morbidity at higher chronological age. Part of this may be due to survivorship bias: those with higher biological age residuals are less likely to live into their 80s and 90s, resulting in an elderly population that is enriched for health-associated traits. This highlights the importance of longitudinal studies across a range of chronological ages to interpret the effects of biological age measures on age-associated outcomes.

As the global population ages, we face an immense burden of age-associated neurological morbidity over the coming decades. Measuring biological age helps us to understand how ageing differs between individuals, and here we have shown that advanced biological age increases the risk of clinically relevant pathology in the nervous system several years into the future. Ultimately this could give a window for putative interventions that slow biological ageing to delay the onset of these disorders.

## Supplementary Material

awad252_Supplementary_Data

## Data Availability

The SATSA cohort has been archived through NACDA Program on Aging (https://www.icpsr.umich.edu/icpsrweb/ICPSR/studies/3843) and detailed information on the study can be found on the Maelstrom Research platform (https://www.maelstrom-research.org/mica/individual-study/satsa). Data archiving is completed for in-person testing waves 1 to 7 and is a work in progress for waves 8 to 10. In addition, all methylation array data are available in the Array Express database of EMBL-EBL (www.ebi.ac.uk/arrayexpress) under the accession number of E-MTAB-7309.
