# Supplementary material for: Advanced biological ageing predicts future risk for neurological diagnoses and clinical examination findings
Source: Brain. 2023 Jul 25;146(12):4891–902. doi: 10.1093/brain/awad252 (PMC10690013; doi:10.1093/brain/awad252)
Supplement: awad252_Supplementary_Data [file awad252_supplementary_data.pdf]

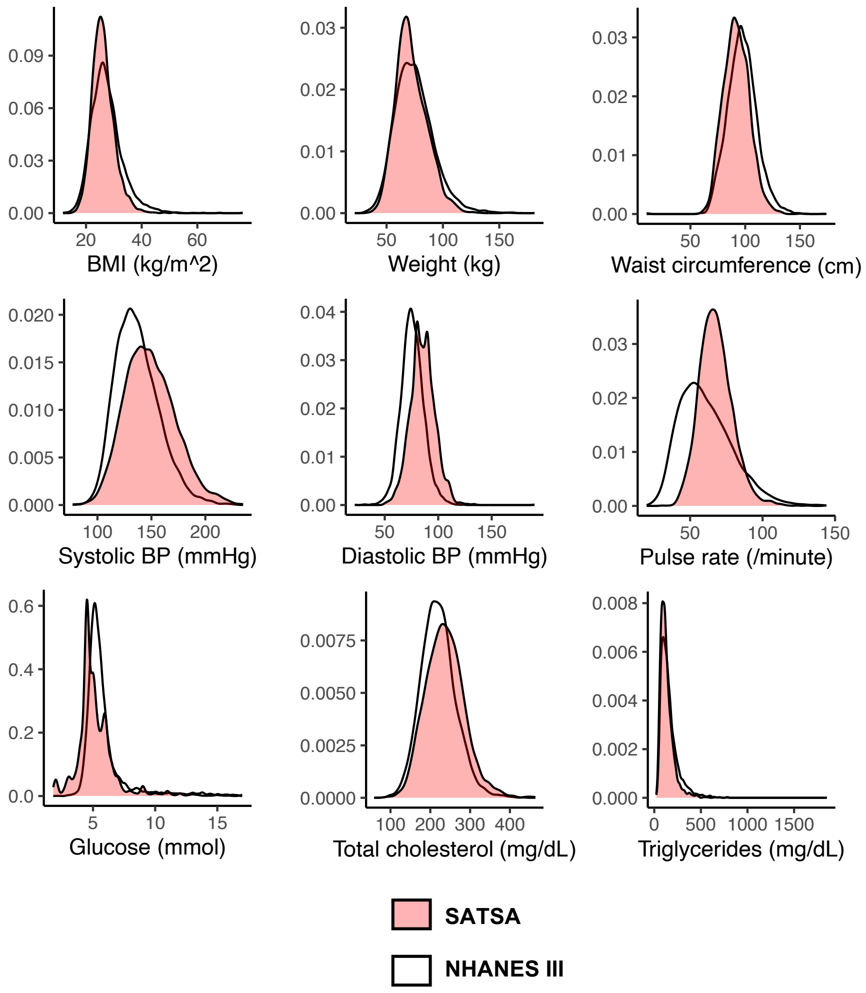

Supplementary Figure 1: Density plots of individual biomarkers in SATSA (pink) compared to NHANES III (white). Data from NHANES III are shown for individuals with a chronological age >45 for better comparison with the population of SATSA, which has a minimum chronological age of 44.9. BMI = body mass index, BP = blood pressure, SATSA = Swedish Adoption/Twin Study of Aging, NHANES = National Health and Nutrition Examination Survey.

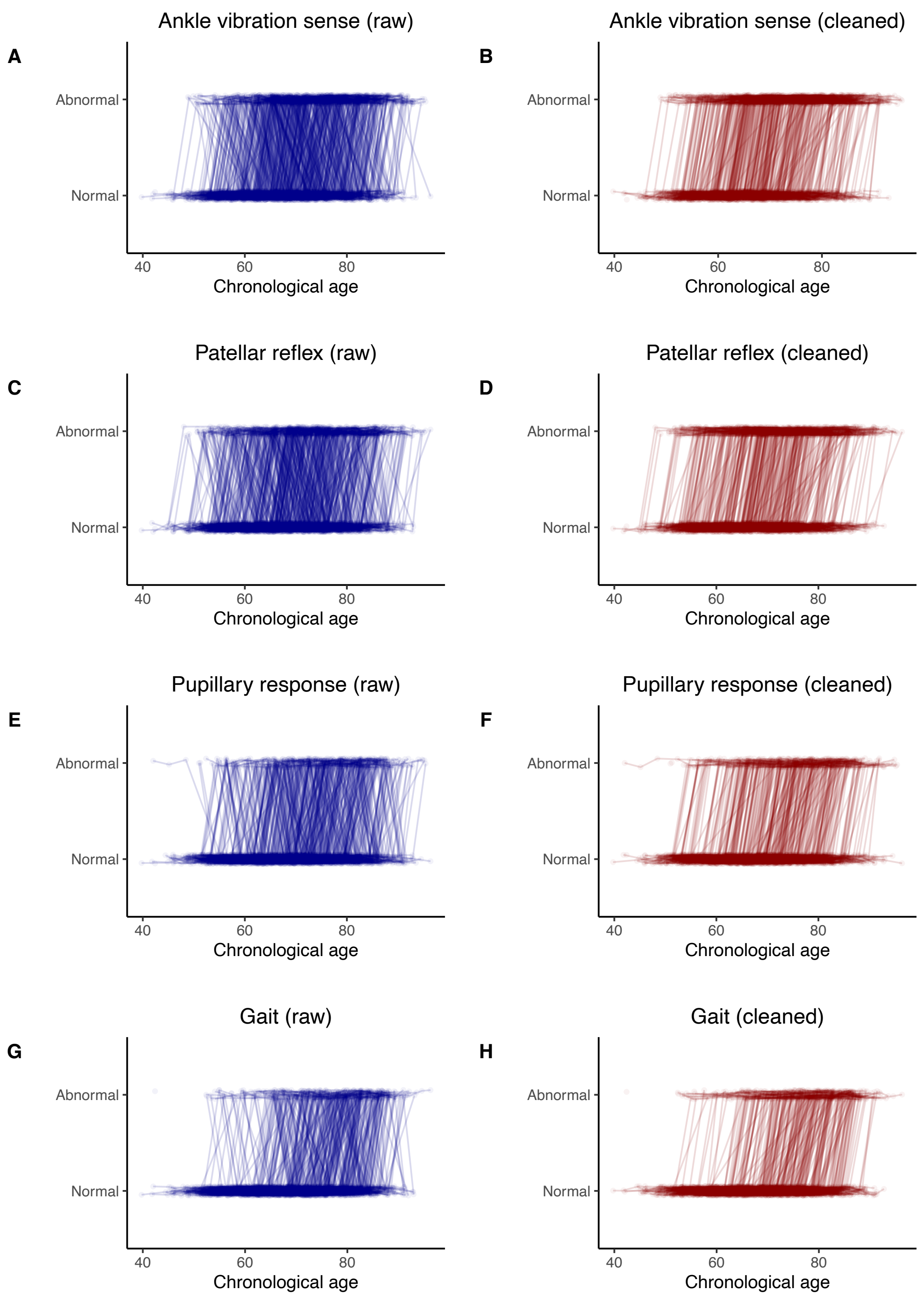

Supplementary Figure 2: Visualisation of the change in age-associated neurological signs with chronological age. The raw data (**A, C, E, G**) and cleaned data, in which abnormal assessments followed by two or more normal assessments are discounted (**B, D, F, H**), are both shown. Each point represents an individual assessment, with longitudinal assessments from the same individual joined by a line. A vertical jitter is applied to better visualise the individual trajectories.

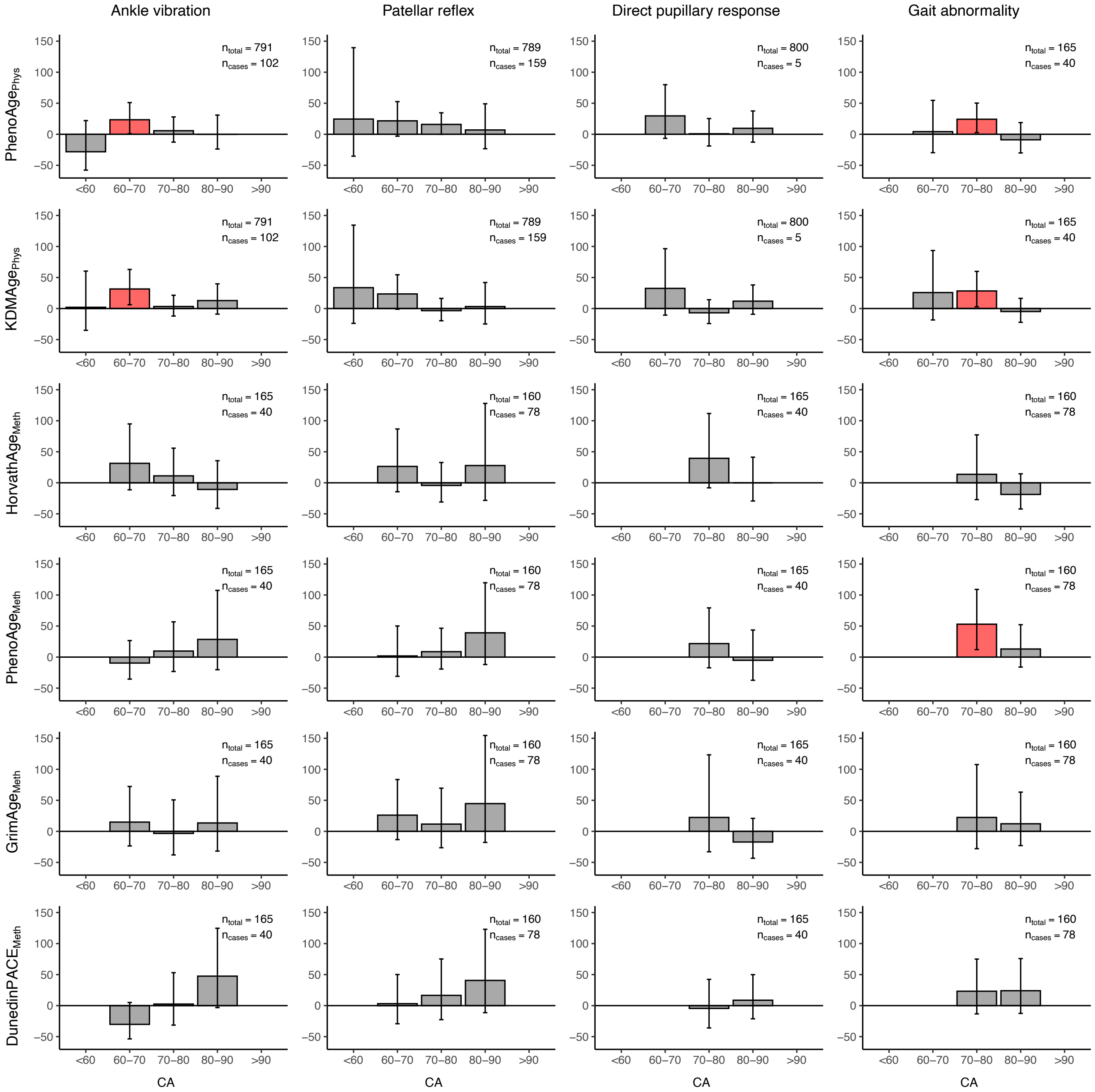

Supplementary Figure 3: Effect of BA residual on risk of developing abnormal neurological signs at different chronological ages. The percentage increases in hazard associated with a 1 standard deviation increase in each BA residual are shown, split by chronological age group for loss of vibration sense at the ankle, development of an abnormal patellar tendon reflex, loss of a normal direct pupillary reflex response to light and development of a gait abnormality. The numbers of individuals in each analysis are reported on the plots. Bars are coloured red for a positive effect and grey for no significant effect on hazard. BA = biological age, CA = chronological age, n_{total} = total population at risk, n_{cases} = number of cases during follow-up.

Supplementary Table 1: Hazard ratios for neurological diagnoses of a 1 standard deviation increase in baseline BA residual for all individuals with a complete set of BA assessments. The total number of individuals at risk (n_{total}) and cases (n_{cases}) contributing to each analysis are shown. Results are displayed as hazard ratio (95% confidence interval) and statistically significant results ($p < 0.05$) are shown in bold.

	Ischaemic stroke	Dementia	Parkinson's disease
n_{total}	358	355	365
n_{cases}	52	83	4
$\text{PhenoAge}_{\text{Phys}}$	1.28 (0.94, 1.74)	0.91 (0.68, 1.22)	1.87 (0.93, 3.75)
$\text{KDMAge}_{\text{Phys}}$	1.24 (0.92, 1.66)	0.86 (0.67, 1.09)	**2.12 (1.48, 3.04)**
$\text{HorvathAge}_{\text{Meth}}$	1.16 (0.87, 1.56)	**1.30 (1.07, 1.57)**	0.97 (0.37, 2.56)
$\text{PhenoAge}_{\text{Meth}}$	1.15 (0.80, 1.65)	1.18 (0.92, 1.51)	1.19 (0.69, 2.04)
$\text{GrimAge}_{\text{Meth}}$	1.41 (0.93, 2.12)	**1.36 (1.01, 1.85)**	1.07 (0.24, 4.80)
$\text{DunedinPACE}_{\text{Meth}}$	**1.30 (1.00, 1.70)**	1.16 (0.93, 1.46)	1.99 (0.82, 4.87)

Supplementary Table 2: Hazard ratios for neurological diagnoses of a 1 standard deviation increase in baseline BA residual for all individuals with physiological BA assessments.

	Ischaemic stroke	Dementia	Parkinson's disease
n_{total}	793	791	800
n_{cases}	109	168	13
$\text{PhenoAge}_{\text{Phys}}$	**1.29 (1.06, 1.58)**	1.09 (0.91, 1.32)	1.01 (0.58, 1.74)
$\text{KDMAge}_{\text{Phys}}$	**1.43 (1.18, 1.73)**	0.98 (0.83, 1.15)	1.03 (0.59, 1.78)

Supplementary Table 3: Hazard ratios for neurological diagnoses of a 1 standard deviation increase in latest premorbid BA residual for all individuals with a complete set of BA assessments.

	Ischaemic stroke	Dementia	Parkinson's disease
n_{total}	331	346	294
n_{cases}	52	83	4
$PhenoAge_{Phys}$	**1.33 (1.07, 1.66)**	0.89 (0.67, 1.17)	1.59 (0.95, 2.65)
$KDMAge_{Phys}$	1.26 (0.97, 1.64)	0.92 (0.73, 1.17)	1.34 (0.88, 2.04)
$HorvathAge_{Meth}$	**1.48 (1.01, 2.15)**	**1.27 (1.03, 1.57)**	1.24 (0.41, 3.73)
$PhenoAge_{Meth}$	1.27 (0.90, 1.79)	**1.28 (1.00, 1.64)**	1.53 (0.40, 5.85)
$GrimAge_{Meth}$	1.39 (0.95, 2.02)	1.29 (0.95, 1.74)	2.89 (0.74, 11.28)
$DunedinPACE_{Meth}$	**1.34 (1.01, 1.78)**	1.15 (0.87, 1.53)	**3.44 (1.07, 11.05)**

Supplementary Table 4: Hazard ratios for neurological diagnoses of a 1 standard deviation increase in latest premorbid BA residual for all individuals with physiological BA assessments.

	Ischaemic stroke	Dementia	Parkinson's disease
n_{total}	756	777	736
n_{cases}	109	168	13
$PhenoAge_{Phys}$	**1.32 (1.10, 1.57)**	0.98 (0.82, 1.18)	0.84 (0.49, 1.44)
$KDMAge_{Phys}$	**1.27 (1.05, 1.53)**	0.97 (0.82, 1.15)	0.71 (0.44, 1.16)

Supplementary Table 5: Hazard ratios for neurological diagnoses of a 1 standard deviation increase in the rate of change of BA between baseline and latest premorbid assessments, for individuals with multiple premorbid complete sets of BA assessments. The number of individuals with Parkinson's disease and multiple premorbid complete BA assessments were too few for the model to generate estimates.

	Ischaemic stroke	Dementia
n_{total}	211	233
n_{cases}	29	48
$PhenoAge_{Phys}$	0.81 (0.57, 1.14)	1.08 (0.67, 1.76)
$KDMAge_{Phys}$	0.81 (0.54, 1.21)	1.07 (0.71, 1.63)
$HorvathAge_{Meth}$	0.89 (0.61, 1.31)	0.96 (0.60, 1.53)
$PhenoAge_{Meth}$	0.84 (0.61, 1.17)	1.09 (0.76, 1.57)
$GrimAge_{Meth}$	0.79 (0.57, 1.09)	0.93 (0.62, 1.39)
$DunedinPACE_{Meth}$	0.84 (0.59, 1.21)	1.00 (0.70, 1.44)

Supplementary Table 6: Hazard ratios for neurological diagnoses of a 1 standard deviation increase in the rate of change of BA between baseline and latest premorbid assessments, for individuals with multiple premorbid sets of physiological BA assessments.

	Ischaemic stroke	Dementia	Parkinson's disease
n_{total}	621	651	590
n_{cases}	90	133	11
$PhenoAge_{Phys}$	0.96 (0.81, 1.14)	1.05 (0.88, 1.26)	1.18 (0.68, 2.05)
$KDMAge_{Phys}$	0.90 (0.73, 1.11)	1.06 (0.87, 1.29)	1.10 (0.75, 1.62)

Supplementary Table 7: Hazard ratios for neurological diagnoses of a 1 standard deviation increase in baseline BA residual for individuals with a complete set of BAs assessments, stratified by twin pairs. The total number of individuals at risk (n_{total}) and cases (n_{cases}) contributing to each analysis are shown. Results are displayed as hazard ratio (95% confidence interval) and statistically significant results ($p < 0.05$) are shown in bold. The number of twin pairs with Parkinson's disease were too few for the model to generate estimates.

	Ischaemic stroke	Dementia
n_{total}	66	88
n_{cases}	37	56
PhenoAge$_{Phys}$	2.50 (0.77, 8.12)	0.90 (0.39, 2.09)
KDMAge$_{Phys}$	1.42 (0.65, 3.08)	1.49 (0.68, 3.30)
HorvathAge$_{Meth}$	1.47 (0.65, 3.33)	1.50 (0.69, 3.26)
PhenoAge$_{Meth}$	1.67 (0.74, 3.77)	1.11 (0.57, 2.17)
GrimAge$_{Meth}$	1.60 (0.45, 5.66)	3.12 (0.94, 10.39)
DunedinPACE$_{Meth}$	1.39 (0.50, 3.84)	0.63 (0.30, 1.33)

Supplementary Table 8: Hazard ratios for neurological diagnoses of a 1 standard deviation increase in baseline BA residual for individuals with baseline physiological BA assessments, stratified by twin pairs.

	Ischaemic stroke	Dementia
n_{total}	178	250
n_{cases}	102	153
PhenoAge$_{Phys}$	1.59 (0.93, 2.71)	1.05 (0.70, 1.57)
KDMAge$_{Phys}$	**1.97 (1.18, 3.27)**	1.09 (0.72, 1.66)

Supplementary Table 9: Lifetime prevalence of abnormal neurological signs on clinical examination amongst SATSA participants.

	n_{cases} (%)
Impaired ankle vibration sense	498 (62.1%)
Abnormal patellar reflex	393 (49.0%)
Impaired pupillary light response	269 (33.5%)
Abnormal gait	277 (34.5%)

Supplementary Table 10: Hazard ratios for abnormal clinical signs of a 1 standard deviation increase in baseline BA residual for all individuals with a complete set of BA assessments. The total number of individuals at risk (n_{total}) and cases with abnormal signs (n_{cases}) contributing to each analysis are shown. Results are displayed as hazard ratio (95% confidence interval) and statistically significant results ($p < 0.05$) are shown in bold.

	Ankle vibration	Patellar reflex	Pupil light reflex	Gait
n_{total}	222	234	273	269
n_{cases}	119	99	94	102
$PhenoAge_{Phys}$	1.22 (0.95, 1.57)	1.18 (0.88, 1.57)	1.11 (0.94, 1.32)	**1.25 (1.03, 1.53)**
$KDMAge_{Phys}$	1.09 (0.88, 1.35)	1.05 (0.83, 1.34)	1.13 (0.98, 1.31)	1.14 (0.91, 1.44)
$HorvathAge_{Meth}$	0.97 (0.76, 1.23)	0.95 (0.76, 1.18)	1.04 (0.80, 1.35)	0.99 (0.78, 1.25)
$PhenoAge_{Meth}$	0.98 (0.78, 1.23)	1.13 (0.87, 1.46)	1.02 (0.79, 1.32)	1.26 (0.99, 1.61)
$GrimAge_{Meth}$	0.88 (0.62, 1.25)	1.09 (0.74, 1.60)	1.13 (0.82, 1.54)	1.09 (0.72, 1.63)
$DunedinPACE_{Meth}$	0.85 (0.67, 1.09)	1.08 (0.86, 1.35)	1.04 (0.81, 1.35)	**1.25 (1.02, 1.53)**

Supplementary Table 11: Hazard ratios for abnormal clinical signs of a 1 standard deviation increase in baseline BA residual for all individuals with physiological BA assessments.

	Ankle vibration	Patellar reflex	Pupil light reflex	Gait
n_{total}	577	628	673	658
n_{cases}	330	289	221	208
$PhenoAge_{Phys}$	1.08 (0.93, 1.24)	**1.19 (1.03, 1.37)**	**1.26 (1.07, 1.48)**	**1.37 (1.17, 1.59)**
$KDMAge_{Phys}$	1.12 (0.98, 1.28)	0.98 (0.86, 1.12)	**1.21 (1.05, 1.40)**	**1.18 (1.00, 1.40)**

Supplementary Table 12: Hazard ratios for abnormal clinical signs of a 1 standard deviation increase in latest premorbid BA residual for all individuals with a complete set of BA assessments.

	Ankle vibration	Patellar reflex	Pupil light reflex	Gait
n_{total}	160	153	166	164
n_{cases}	119	99	94	102
$PhenoAge_{Phys}$	1.08 (0.83, 1.41)	1.08 (0.81, 1.45)	1.15 (0.91, 1.45)	**1.22 (1.02, 1.45)**
$KDMAge_{Phys}$	0.86 (0.67, 1.11)	0.98 (0.78, 1.25)	0.99 (0.83, 1.17)	1.13 (0.91, 1.39)
$HorvathAge_{Meth}$	1.15 (0.96, 1.37)	1.00 (0.82, 1.21)	**1.29 (1.06, 1.58)**	1.15 (0.91, 1.44)
$PhenoAge_{Meth}$	1.03 (0.86, 1.22)	1.04 (0.87, 1.26)	1.03 (0.81, 1.32)	**1.24 (1.03, 1.49)**
$GrimAge_{Meth}$	0.94 (0.75, 1.18)	1.15 (0.90, 1.48)	1.28 (0.93, 1.76)	**1.31 (1.01, 1.70)**
$DunedinPACE_{Meth}$	1.00 (0.79, 1.26)	1.14 (0.91, 1.43)	0.98 (0.78, 1.24)	1.10 (0.88, 1.39)

Supplementary Table 13: Hazard ratios for abnormal clinical signs of a 1 standard deviation increase in latest premorbid BA residual for all individuals with premorbid physiological BA assessments.

	Ankle vibration	Patellar reflex	Pupil light reflex	Gait
n_{total}	440	457	395	343
n_{cases}	330	289	221	208
$PhenoAge_{Phys}$	**1.15 (1.00, 1.32)**	**1.17 (1.06, 1.29)**	1.12 (0.97, 1.29)	1.11 (0.92, 1.34)
$KDMAge_{Phys}$	1.01 (0.89, 1.14)	0.98 (0.87, 1.10)	1.02 (0.89, 1.17)	1.01 (0.88, 1.17)

Supplementary Table 14: Hazard ratios for abnormal clinical signs of a 1 standard deviation increase in the rate of change of BA between baseline and latest premorbid assessments, for individuals with multiple premorbid complete sets of BA assessments.

	Ankle vibration	Patellar reflex	Pupil light reflex	Gait
n_{total}	89	80	72	81
n_{cases}	74	58	45	52
$PhenoAge_{Phys}$	1.13 (0.87, 1.48)	1.10 (0.73, 1.66)	0.84 (0.62, 1.14)	0.80 (0.61, 1.05)
$KDMAge_{Phys}$	0.96 (0.76, 1.21)	1.04 (0.70, 1.54)	0.86 (0.64, 1.16)	0.96 (0.71, 1.28)
$HorvathAge_{Meth}$	0.94 (0.77, 1.16)	0.89 (0.68, 1.16)	1.16 (0.87, 1.55)	0.97 (0.77, 1.24)
$PhenoAge_{Meth}$	1.12 (0.88, 1.43)	1.07 (0.77, 1.50)	0.86 (0.63, 1.18)	0.87 (0.70, 1.09)
$GrimAge_{Meth}$	0.91 (0.70, 1.17)	0.86 (0.59, 1.25)	1.28 (0.85, 1.92)	1.25 (0.83, 1.90)
$DunedinPACE_{Meth}$	0.96 (0.73, 1.26)	0.79 (0.58, 1.06)	1.10 (0.87, 1.38)	0.86 (0.66, 1.11)

Supplementary Table 15: Hazard ratios for abnormal clinical signs of a 1 standard deviation increase in the rate of change of BA between baseline and latest premorbid assessments, for individuals with multiple premorbid sets of physiological BA assessments.

	Ankle vibration	Patellar reflex	Pupil light reflex	Gait
n_{total}	303	282	295	280
n_{cases}	219	159	168	177
$PhenoAge_{Phys}$	1.12 (0.93, 1.36)	1.11 (0.90, 1.36)	0.92 (0.73, 1.16)	**0.79 (0.67, 0.92)**
$KDMAge_{Phys}$	0.96 (0.85, 1.09)	1.15 (0.93, 1.41)	0.84 (0.68, 1.03)	0.90 (0.76, 1.08)

Supplementary Table 16: Hazard ratios for abnormal clinical signs of a 1 standard deviation increase in baseline BA residual for individuals with a complete set of baseline BA assessments, stratified by twin pairs. The total number of individuals at risk (n_{total}) and cases (n_{cases}) contributing to each analysis are shown. Results are displayed as hazard ratio (95% confidence interval) and statistically significant results ($p < 0.05$) are shown in bold.

	Ankle vibration	Patellar reflex	Pupil light reflex	Gait
n_{total}	130	98	102	110
n_{cases}	87	63	63	76
PhenoAge$_{Phys}$	0.99 (0.49, 2.00)	0.90 (0.36, 2.20)	1.56 (0.64, 3.76)	1.09 (0.44, 2.70)
KDMAge$_{Phys}$	1.34 (0.57, 3.15)	0.97 (0.31, 3.02)	1.35 (0.52, 3.51)	0.84 (0.34, 2.05)
HorvathAge$_{Meth}$	1.36 (0.68, 2.72)	1.25 (0.41, 3.76)	1.69 (0.63, 4.54)	1.02 (0.29, 3.61)
PhenoAge$_{Meth}$	1.03 (0.56, 1.89)	1.45 (0.38, 5.56)	0.79 (0.33, 1.91)	0.76 (0.20, 2.85)
GrimAge$_{Meth}$	0.65 (0.28, 1.51)	0.93 (0.27, 3.15)	1.43 (0.56, 3.62)	0.44 (0.08, 2.44)
DunedinPACE$_{Meth}$	0.80 (0.46, 1.36)	0.71 (0.18, 2.81)	0.92 (0.53, 1.61)	0.94 (0.41, 2.14)

Supplementary Table 17: Hazard ratios for abnormal clinical signs of a 1 standard deviation increase in baseline BA residual for individuals with baseline physiological BA assessments, stratified by twin pairs.

	Ankle vibration	Patellar reflex	Pupil light reflex	Gait
n_{total}	358	342	290	274
n_{cases}	249	229	180	173
PhenoAge$_{Phys}$	0.98 (0.66, 1.46)	1.05 (0.77, 1.45)	1.35 (0.86, 2.10)	0.94 (0.63, 1.42)
KDMAge$_{Phys}$	1.10 (0.76, 1.60)	1.10 (0.78, 1.56)	1.03 (0.70, 1.50)	0.84 (0.54, 1.31)
